# Teachers and school administrators’ experiences with professional development feedback: The classroom strategies assessment system implementation

**DOI:** 10.3389/fpsyg.2023.1074278

**Published:** 2023-02-23

**Authors:** Maria S. Poulou, Linda A. Reddy, Christopher M. Dudek

**Affiliations:** ^1^Department of Educational Sciences and Early Childhood Education, University of Patras, Patras, Greece; ^2^Graduate School of Applied and Professional Psychology, Rutgers University, New Brunswick, NJ, United States

**Keywords:** observational assessment, elementary teachers, instructional and behavior management practices, professional development, performance feedback

## Abstract

To support teachers’ evaluation and professional development valid assessments that measure teachers’ classroom practices and capture teachers’ strengths and areas in need of improvement are needed. The current study examined school administrators’ and teachers’ experiences of Professional Development (PD), with the use of the Classroom Strategies Assessment System (CSAS), a classroom observational assessment that measures universal classroom instructional and behavioral practices, and their perceptions of the usability of CSAS for supporting PD. The study also examined school administrators’ ratings of elementary school teachers’ use of evidence-based instructional and behavior management practices, as an illustrative example of how performance feedback was implemented. Three school administrators observed 31 elementary school teachers three times each using the CSAS Greek version. Following each observation, teachers received brief performance feedback based on CSAS scores from their school administrator. School administrators and teachers completed the System Usability Scale to assess the usability of the CSAS administration. Semi-structured interviews with 19 of the participating teachers were conducted to further explore teachers’ professional development experiences. Overall, teacher interviews expressed their need for professional development in the areas of instructional and behavior management practices and perceived CSAS feedback helpful for instructional improvement. Findings also suggest some improvements in the frequency and quality of teacher instructional and behavior management practices as measured by the CSAS. Implications for practices and research are discussed.

## Introduction

Effective instructional and behavioral management classroom practices have been well documented for more than 50 years of effective teaching research (Simonsen et al., 2008). Yet, every day more than 3.9 million teachers in the United States and millions of others around the world face challenges educating students with diverse needs, knowledge, and skills (Kraft et al., 2018). Internationally, policy makers and education systems are concerned with the increasing rates of student disengagement (Hepburn and Beamish, 2019), student emotional and behavioral difficulties (Armstrong, 2018), and teachers leaving the profession due to classroom management problems (Darling-Hammond et al., 2017; Dudek et al., 2018; Fabiano et al., 2018; Reddy et al., 2018a,b). At the same time research underscores gaps between effective universal teaching practices and their implementation in classrooms (Reinke et al., 2012; Simonsen et al., 2019; Poulou et al., 2020). One way to reduce the gap between research and practice is through integrating and supporting the use of evidence-based practices in the learning contexts they were designed for (Cappella et al., 2011). Teachers need effective teaching strategies and ongoing professional development (PD) support to increase their implementation of effective practices (Simonsen et al., 2019). The current study suggests instructional coaching embedded with performance feedback as an effective form of PD that can provide a promising method for supporting teachers’ implementation of classroom practices and bridging the implementation gap.

### Teacher performance feedback

The failure of traditional professional development (PD) programs to improve teachers’ instructional demands (Desimone, 2009) has shifted research interests towards the application of on-the-job PD for teachers. In general, research has found job-embedded approaches to offer large potential for teacher education (Kraft et al., 2018). Combining traditional lecture with hands-on practice and feedback ameliorates the knowledge and skill transfer limitations of workshop-only based PD (Joyce and Showers, 2002). These types of school-based PD for teachers increases the likelihood that teachers engage in evidence-based classroom management strategies (Sugai and Horner, 2006).

Recently, instructional coaching has emerged as a promising method of PD, where a teacher works with an expert to learn new practices. The goal of coaching is to provide non-evaluative feedback to teachers by emphasizing on teacher’s awareness of their teaching practices (Sutherland, 2000) and opportunities for reflection based on observation data (Stitcher et al., 2009). Instructional coaching was found to be linked with large positive effects of coaching on teacher practices and meaningful improvements in student achievement (Kraft et al., 2018).

Effective PD programs, like instructional coaching, should include opportunities for performance feedback (Darling-Hammond et al., 2017). Performance feedback is an evidence-based intervention (Fallon et al., 2015), which entails objective information regarding teacher’s instruction, direct feedback on the implementation of practices, and action plans to improve implementation. It is otherwise called “knowledge of the results,” where the data generated in the performance observation is provided to the observed performer (Kluger and DeNisi, 1996). There are differences in terms of the delivery of performance feedback (Codding et al., 2008; Auld et al., 2010; Myers et al., 2011) and the information presented (Ford, 1984; Alvero et al., 2001; Reinke et al., 2007). A review of studies concludes that it is unclear which elements of performance feedback are critical and whether these vary based on the implementer (Fallon et al., 2015; Luck et al., 2018). However, given the small number of studies employing various forms of performance feedback, it is difficult to discern which form of feedback may be most effective for promoting practices (Cavanaugh, 2013).

Teacher coaching with performance feedback and specifically task-specific feedback rather than person-focused feedback (Kluger and DeNisi, 1996) has been found to be effective for improving teachers’ use of behavior-specific praise (Cavanaugh, 2013), opportunities to respond (Cavanaugh, 2013), better communication with students (Rathel et al., 2008) and effective strategy usage (Stitcher et al., 2009), Furthermore, in order to enhance uptake and skill transfer into the classroom, it is important that teacher evaluators’ and teachers’ perceptions of PD interventions and implementation approaches are considered. Teachers’ social validity, that is the acceptability and satisfaction with the evaluators and the implementation process consists of a prerequisite of implementation efforts (Fowler, 2004), whereas failure to address teachers’ perspectives is a threat to the implementation fidelity (Gresham et al., 1993).

Although there is agreement that coaches need to implement and evaluate PD tailored to individual teacher’s needs and feedback preferences (Simonsen et al., 2019), few studies had examined teacher experiences with classroom observational assessment and evaluation (Reddy et al., 2016). Little is known regarding teachers’ experiences with school-based coaches (Hoagwood et al., 2007). In most of the studies reviewed the training and/or performance feedback was provided by researchers or university student-teacher supervisors (Cavanaugh, 2013). It is unknown therefore whether the results of these studies could be generalized to “natural implementers” (i.e., school administrators, school-based coaches, teachers) who are situated within the school context and provide support and performance feedback to teachers in schools.

### Current study

Research has examined the mechanisms of teacher PD on classroom practices and student educational outcomes for some time (Simonsen et al., 2008; Desimone, 2009; Gilmour et al., 2019). However, the vast majority of investigations have been focused on workshop-based training and limited job-embedded feedback overtime. Likewise, the vast majority of research on the process of teacher performance feedback has been conducted by university researchers, external to schools community (Cavanaugh, 2013; Dudek et al., 2019). Thus, studies are needed that include natural school implementers, school administrators and teachers who are responsible for facilitating schools (Cavanaugh, 2013). At the same time, coaches have few empirically supported tools which support the identification and monitoring of change in teacher instructional and behavioral management practices over time (Reddy et al., 2017; Poulou et al., 2020). Classroom observational assessments are one commonly used approach school administrators and other school personnel (e.g., instructional coaches, peer mentors) use to identify teacher practice strengths and areas in need of improvement (Reddy et al., 2013a; Gilmour et al., 2019).

This void is particularly noted outside of the United States and in particular, the country of Greece. There are very limited research studies on school administrators’ and teachers’ PD experiences (Beazidou et al., 2013; Koutrouba et al., 2018), or the use of teacher observational assessment for PD. In a study of Greek teachers’ attitudes relating to teaching profession, teachers noted the importance of continuing training and learning for their PD in order to keep up with the professional demands and responsibilities (Ifanti and Fotopoulou, 2011).

The current study aims to examine PD experiences for school administrators and teachers in Greece, with the use of classroom observational assessment in enhancing the PD experiences. The current study serves as the first investigation to address the usability of a valid classroom assessment tool translated for elementary schools in Greece, the Classroom Strategies Assessment System (CSAS), which can be used as an example of performance feedback implementation. To address this gap in the literature, the current study addresses the following research questions:

1. Do school administrators and teachers rate the usability of the CSAS Greek version and score performance report positively for informing PD?
2. What are teachers’ experiences with PD feedback in general and the performance feedback they received from the CSAS specifically?
3. Does the assessment of teachers’ use of evidence-based instructional and behavior management practices, as measured by the CSAS, change following brief performance feedback in Greek elementary schools?

## Method

### Participants

Observations were conducted on 31 elementary teachers (elementary teachers teach students aged 6–12 years old), from three public schools in western Greece and four public schools from Chios island. The distribution of teachers by grade assignment were as follows: 7 taught first grade, 7 taught second grade, 2 taught third grade, 6 taught fourth grade, 4 taught fifth grade and 5 taught sixth grade. Teachers were primarily Caucasian females (27 females; over 80%), proficient in Greek and English and their years of teaching experience ranged from 10 to 20 years. All teachers voluntarily participated in the project, which included observations coupled with performance feedback meetings, as well as a semi-structured interviews with questions relating to the performance feedback they received. Three school administrators (two females, one male) conducted the observations. The observers observed teachers three times (with 1–2 weeks interval) for 30 min each, in Greek Language Arts or mathematics. In sum, observers completed 91 CSAS observations (30 teachers were observed 3 times and 1 teacher was observed 1 time) across 3 time points. Observers observed the same teacher, in the same content area.

### Instruments

#### CSAS observer form

The CSAS is a multi-method and multi-rater classroom observational assessment that measures universal classroom instructional and behavioral practices (Reddy et al., 2013b). The CSAS examines and provides feedback on the presence of evidence-based instructional and behavior management strategies found related to student learning (Gable et al., 2009; Moore Partin et al., 2010; Reddy et al., 2013b).

The CSAS-Observer form consists of the Strategy Counts and the Strategy Rating Scales. The Strategy Counts contains 8 strategies (4 based on instruction; 4 based on classroom management) that are discretely counted during classroom observations; observers tally the frequency with which each strategy is used by teachers, either for an individual student or a group of students. The Strategy Rating Scales include the Instructional Strategies (IS) and Behavioral Management Strategies Rating Scales (BMS). The Instructional Strategies Scale includes 28 items, comprised of 5 scales: Adaptive Instruction (4 items), Student-Directed Instruction (5 items), Direct Instruction (8 items), Promotes Student Thinking (5 items), and Academic Performance Feedback (6 items). The Behavioral Management Strategies Scales includes 26 items, categorized into 4 scales: Directives (6 items), Proactive Methods (8 items), Praise (5 items), and Corrective Feedback (7 items).

For both the IS and BMS Rating Scales, observers have to rate each item in terms of the *observed frequency* (how often teachers used the instructional and behavioral management strategies), and the *recommended frequency* (the degree to which these strategies should have been used in the specific classroom context), on a 7-point Likert scale (1 = not used, 7 = always used). The *discrepancy score* is then calculated by taking the absolute value of the difference between the observed frequency and the recommended frequency ratings. An item discrepancy score of zero indicates that teachers’ observed use of strategy matched the recommended use of that strategy, whereas an increase of the discrepancy scores indicates that teachers deviated from the recommended use of strategies. A plethora of studies provide evidence of CSAS’ psychometric properties (Reddy et al., 2013a,b,c; Poulou et al., 2020).

#### System usability scale (SUS)

The SUS was used to determine the usability of the CSAS – Greek Form. For this study we adapted the SUS by Brooke (1996) and its refined version by Bangor et al. (2008). The modified 5-point Likert scale features 10 items (0 = *strongly disagree* to 4 = *strongly agree*), which are multiplied by 2.5 to arrive a total score range of 0 to 100. Moreover, normative and criterion-referenced interpretations of the total score are also available (Finstad, 2006; Bangor et al., 2008). For the current study, the Cronbach alpha for the SUS was 0.67.

#### Teacher interviews

Semi-structured interviews investigated teachers’ perceptions of the following themes: (a) the performance feedback they currently receive for teaching effectiveness (i.e., describe how you receive performance feedback about your teaching experience, what is the form of feedback), (b) needs and anticipations for their teaching improvement (i.e., describe in which teaching domains do you need more support), (c) CSAS performance feedback (i.e., what were your expectations and experiences participating in the study), and (d) suggestions for future PD.

### Procedures

Following the approval of our study by the institutional review board of the first author’s department, the first author and school administrators met with the school directors and the teachers who agreed to participate in the study, in order to explicitly describe the process of observations, feedback meetings, completion of questionnaires, and interviews.

#### CSAS and SUS translation process

The CSAS and the adapted SUS were initially translated into Greek by the lead author and then two bi-lingual English-Greek graduate students from the University of Patras reverse translated the CSAS and SUS Greek versions back into the English to check for accuracy. Next, 5 elementary Greek teachers volunteered to assess word accuracy as well as face/content validity of the measures.

#### Observer training

The three independent observers were trained on the CSAS by a master trainer. Two observers had a Master’s degree and one observer had a doctorate. Collectively, their years of teaching experience ranged from 15 to 25 years. The Greek observers received five didactic training sessions (3 h each) from a CSAS Trainer/Master Coded through webinars.

#### CSAS administration and scoring

Teachers of the study were observed 3 times each (October–December, 2019) using the CSAS-Observer Form and each observation was a total of 30 min in length. Observations occurred during instructional periods in which a variety of instructional activities occurred, such as review of the previous lesson, introduction of new material, whole group instruction, group work, independent practice and checking answers on completed work. Observers sat in an unobtrusive location in the back of the classroom during the observations. During the observations, the observer/coach counted the frequency with which the teacher uses the instructional and behavioral strategies listed in CSAS Strategy Counts described above. Following the observation, the coach rated the recommended and observed frequency of using each strategy listed in Strategy Ratings.

#### Performance feedback meetings

Following each observation, school administrators conducted 3 follow-up feedback sessions with each teacher during school hours. Each session lasted from 15 to 30 min. After each observation, school administrators provided observed teachers with individual visual graphs depicting performance rates in terms of the frequency and the quality of instructional and behavior management strategies (as indicated by CSAS frequency and discrepancy scores) as points of reference for each feedback session. Sessions focused on reviewing and discussing the (1) CSAS score report that included visual performance feedback, (2) areas of practice strengths and areas for considering improvement, (3) contextualized examples of evidence-based practices which could be alternatively used by teachers in the specific classroom observed, and (4) mutual agreement between teachers and school administrators about the way the teacher intended to employ the classroom practices.

#### Teachers’ interviews and qualitative responses

Observed teachers were invited by their school administrators to participate in semi-structured telephone interviews with the lead author. Interviews were recorded and transcribed for thematic analysis to generate themes across participants (Boyatzis, 1998; Braun and Clarke, 2006). The audio-taped interviews were independently coded by three raters trained in the interview protocol. The coding team developed a structured codebook, including definitions of codes, inclusion and exclusion criteria, and example text. Coders came to consensus on the internal coherence and consistency within those subthemes.

## Results

### Research question 1

Do school administrators and teachers rate the usability of the CSAS Greek version and score performance report positively for informing PD?

After the CSAS was administered three times, school administrators and teachers were asked to rate SUS (Table 1). Overall, both types of users rated the CSAS as easy to use (*M* = 4.00, SD = 1.41, for observers and *M* = 3.60, SD = 0.65 for teachers), with clear instructions (*M* = 4.00, SD = 0.00 for observers and *M* = 3.95, SD = 0.56 for teachers). In general, observers viewed the CSAS slightly more favorably than teachers, especially when asked if the CSAS could guide progress in teachers’ PD (*M* = 5.00, SD = 0.00 for observers and *M* = 4.00, SD = 0.67 for teachers). The SUS Total mean scores for all the items for school administrators and teachers were 4.58 (SD = 0.35) and 4.12 (*SD* = 0.64) respectively.

**Table 1:** Usability ratings for CSAS Greek form and reporting by school administrators (observers) and teachers.

	Questions	Observers (*N* = 3)	Teachers (*N* = 23)
		*M* (SD)	*M* (SD)
1.	I think that I could use the CSAS to communicate progress in teachers’ professional development.	5.00 (0.00)	4.00 (0.67)
2.	I found the CSAS unnecessary.	1.00 (0.00)	1.26 (0.54)
3.	I thought the CSAS was easy to use.	4.00 (1.41)	3.60 (0.65)
4.	I found the CSAS scores and graphs as helpful.	4.50 (0.70)	4.00 (0.67)
5.	I liked the CSAS Strategy Counts (tallies).	4.00 (0.00)	4.00 (0.52)
6.	I like the Strategy Rating Scales 7-level ratings of observed frequency and recommended frequency.	4.50 (0.70)	4.21 (0.51)
7.	I like the CSAS feedback reports (graphs).	5.00 (0.00)	4.45 (0.59)
8.	I think the use of the CSAS will have positive consequences on teaching.	4.50 (0.70)	4.26 (0.61)
9.	I could complete the CSAS right after a classroom observation to provide quick and accurate information.	4.50 (0.70)	3.52 (0.89)
10.	I think the CSAS could negatively impact the teaching process.	1.00 (0.00)	1.65 (0.93)
11.	The instructions for using the CSAS were clear.	4.00 (0.00)	3.95 (0.56)
12.	Overall, I really liked using the CSAS feedback report to summarize specific qualities of teaching.	5.00 (0.00)	4.39 (0.58)
	Τotal sum	55.00 (4.21)	49.45 (7.72)
	Total mean	4.58 (0.35)	4.12 (0.64)

### Research question 2

What are teachers’ experiences with PD feedback in general and the performance feedback they received from the CSAS specifically?

Semi-structured interviews with 19 teachers were conducted to investigate teachers’ perceptions of (a) the performance feedback they currently receive for teaching effectiveness; (b) needs and anticipations for their teaching improvement; (c) CSAS performance feedback; and (d) suggestions for future PD.

In regard to current performance feedback, the majority of teachers observed indicated that they did not receive any type of formal performance feedback prior to our study. In the question regarding the support they receive for their PD each year, the majority of them (14/19) received support from training sessions or seminars organized either by school agents or from private sectors. In terms of PD needs and anticipation for improvement, teachers underscored their need in the field of classroom management (10/19), teaching strategies (7/19), self-improvement (5/19), students’ special needs (learning difficulties, gifted children 5/19) and interaction with parents (2/19). For CSAS performance feedback, teachers were first asked about the expectations they had from their participation in the study. The majority of teachers mentioned that they hoped for PD improvement (14/19), an objective picture of their teaching (5/19), and concrete feedback on instructional and behavior management practices (3/19). Teachers were next asked to describe the experience of their participation in the study. All of them reported that the experience was very interesting and constructive (e.g., “I received specific feedback that improved my instruction,” “data that informed my classroom practices”). Teachers admitted that the feedback they received was very focused, explicit, immediate, brief, and informative about the teaching strategies which they had to ameliorate, as well as suggestions to achieve this. All teachers perceived the performance feedback with the CSAS very helpful, having them to reflect on their teaching. Teachers also mentioned that the feedback they received mainly helped the way they implemented instructional strategies, in comparison to behavior management strategies. Lastly, teachers were asked to make suggestions for future PD. All but one teacher agreed that the use of CSAS would be a helpful tool for their PD. Teachers were asked whether classroom observations would serve as a helpful method for performance feedback. Eleven teachers admitted that observations from colleagues would provide support and feedback providing a climate of support and confidentiality with no evaluative purpose. Finally, teachers suggested a variety of models to support their teaching effectiveness such as: meeting with colleagues (reported by 5 teachers), meetings with community agents (reported by 3 teachers), monthly meetings with school administrator (reported by 1 teacher), assessment by colleagues and school administrators (reported by 3 teachers), modelling teaching by school administrators or university teachers (reported by 2 teachers) and finally workshops (reported by 7 teachers).

### Research question 3

Does the assessment of teachers’ use of evidence-based instructional and behavior management practices as measured by the CSAS, change following brief performance feedback in Greek elementary schools?

Table 2 presents the means and standard deviations of the CSAS scores for each of the three observations. For Strategy Counts, Academic Response Opportunities and Academic Praise were the most frequently used instructional practices. The frequency of discrete instructional and behavior management strategies was comparable across observations with the exception of Academic Praise and Vague Directives which increased over time. Repeated measures ANOVA for observations 1 and 3 revealed significant increases in Academic Praise (*F* = 6.35, df = 1, *p* = 0.01; effect size of 0.80) and Vague Directives (*F* = 4.23, df = 1, *p* = 0.04, effect size of 0.76) across observations.

**Table 2:** Means and standard deviations of strategy counts (frequency) and strategy rating scales (discrepancy scores).

	Observation 1		Observation 2		Observation 3		Effect-sizes^a^
	M	SD	M	SD	M	SD	
Strategy counts total	6.65	5.72	6.26	6.43	6.82	6.92	0.02
Concept summaries	5.74	5.37	6.43	6.07	6.00	6.49	0.04
Academic praise	10.13	6.60	12.80	8.42	15.47	11.39	0.80^b^
Academic response opportunities	24.52	16.95	27.73	18.07	26.70	13.01	0.12
Academic corrective feedback	7.87	5.87	6.07	3.18	6.60	3.86	0.21
Clear directives	6.58	4.38	6.63	6.71	7.63	5.88	0.23
Behavioral praise	1.52	2.24	1.03	2.12	1.03	2.65	0.21
Vague directives	0.68	1.42	1.50	3.06	1.77	3.42	0.76^b^
Behavioral corrective feedback	8.97	8.68	7.70	6.29	7.53	7.45	0.16
							
Strategy rating scales total (IS + BMS) score	40.58	17.12	32.30	13.00	34.33	18.85	0.36
							
Instructional strategies (IS) total	18.39	9.46	14.73	6.91	15.47	10.72	0.30
Adaptive instruction	2.26	1.86	1.70	1.36	2.30	2.18	0.02
Student-directed Instruction	4.06	2.83	3.60	3.06	4.27	3.46	0.07
Direct instruction	3.81	3.66	3.00	3.00	2.93	3.27	0.24
Promotes students’ thinking	4.16	2.05	3.33	2.32	3.17	2.39	0.48^b^
Academic performance feedback	4.10	3.25	3.10	2.18	2.80	2.79	0.40

Behavioral management strategies (BMS) total	22.19	9.45	17.57	7.56	18.87	9.13	0.35
Directives	3.00	2.85	2.03	1.97	2.30	314	0.24
Proactive methods	4.61	3.57	3.50	3.85	2.93	3.05	0.47
Praise	10.77	5.66	9.50	4.64	11.87	5.25	0.19
Corrective feedback	3.81	3.08	2.53	3.06	1.77	3.11	0.66^b^

a Within group effect size between observations 1 and 3.

b Indicates repeated measures ANOVA F tests computed between observations 1 and 3 revealed significant differences *p* < 0.05.

Table 2 presents the means, standard deviations for each of the three observations for the Strategy Rating Scales. Strategy Rating Scales present the discrepancy scores between the observed frequency and recommended frequency, denoting that small discrepancy scores indicate greater quality use of classroom strategies. For the IS Rating Scales, the IS Total and subscales of Direct Instruction, Promotes Students’ Thinking and Academic Performance Feedback reflected a gradual decrease (improvement) in discrepancy scores from observations 1 to 3. For the BMS Rating Scales, the Proactive Methods and Corrective Feedback subscales presented a gradual decrease (improvement) in discrepancy scores from observations 1 to 3. ANOVA revealed significant improvements in the Promoting Student Thinking subscale (*F* = 4.51, df = 1, *p* = 0.04, effect size of 0.48), and the Behavioral Corrective Feedback subscale (*F* = 12.01, df = 1, *p* = 0.00, effect size of 0.66). Overall effect sizes were small to moderate for Strategy Counts (0.04–0.76), small for IS Strategy Rating Scales (0.02–0.48), and small to moderate for Behavior Management Rating Scales (0.24–0.66; Cohen, 1988).

## Discussion

The current study serves as the first investigation of school administrator’s use of observational assessment feedback using the CSAS outside United States. We first examined the usability of the CSAS for informing PD, using school administrators’ and teachers’ ratings on the SUS Greek version. Teachers and mainly teacher administrators gave high preference rates for the CSAS measure and its usefulness for teachers’ support on instructional and behavior management practices. The semi-structured interviews conducted with teachers added to the limited literature that explores teachers’ perceptions of and experiences with PD programs on instructional and behavior management strategies in Greece and offer insights into the benefits of assessment-driven performance feedback on universal practices for teachers. Likewise, we examined school administrator’s use of observational data to inform changes in elementary school teachers’ use of universal classroom practices in Greece. Findings in general highlight some changes in teacher practices following iterative cycles of observations and brief feedback. Specifically, changes in practices were observed in the frequency of using Academic Praise and Vague Directives and in the quality of Promote Students’ Thinking and Corrective Feedback implementation.

### Usability of CSAS Greek version

Following the third observation, school administrators and teachers completed a social validity measure, the Usability Ratings for CSAS-Greek form. Results were positive, indicating that teachers and mainly school administrators were satisfied with the use of CSAS process, the clarity of instructions, the feedback report graphs and the positive consequences for teaching. Both teacher and school administrators reported their preference in using the CSAS feedback report to emphasize on qualities of teaching. Ratings on the Usability Ratings for CSAS-Greek form are similar to usability findings on the CSAS-English form in a study by Fabiano et al. (2018), which suggests that the CSAS may be a valuable tool to provide individualized teacher feedback and follow-up support based on teacher’s contextualized needs (Reddy et al., 2017).

### Teacher experience with PD feedback

Teacher’ perspectives were featured in this initial investigation, thus providing social validity to our study. The current study suggests that performance feedback could be a promising type of PD as long as it is not used for evaluative purpose. Although different types of training and feedback are needed for teachers based on their experience, self-efficacy, or motivation to change (Cavanaugh, 2013), teachers in our study indicated that they preferred brief and immediate feedback reports following the classroom observations. Literature on performance feedback also underlines the importance of immediacy in providing feedback to teachers (Scheeler et al., 2004).

Teachers of our study suggested peers have the role of observers, providing a climate of trust and confidentiality. Similarly in another Greek study of secondary teachers’ perceptions of adult learning, researchers concluded that the apparent inferiority when teachers are compared with academic tutors bolster the issue of “them” and “us,” which consists of a detrimental factor for professional progress (Gravani and John, 2005). In fact, the use of regular, non-evaluative feedback may be a more effective tool for promoting teaching performance (Knight, 2007). Teacher’s perceptions of peer affiliation is a promising finding for understanding positive changes in teacher classroom behavior and student outcomes (Sebastian et al., 2019). When people do not feel they are judged by peers or supervisors, professional learning can actually take place (Brinko, 1993). Further research needs to investigate how personal variables between the coach and the teacher, such as trust and positive communication impact the effectiveness of the feedback.

Finally, teachers in our study suggested workshops as a type of PD. Given the poor effects of workshop training alone (Fixsen et al., 2005), we need to familiarize teachers with forms of PD, equally brief, cost-effective, which could provide effective and sustained professional experiences on teaching and classroom management strategies (Oliver and Reschly, 2007). Performance feedback with CSAS was found to be a helpful suggestion aligned with teachers’ needs on instructional and behavior management practices.

### School administrators’ assessment of teacher practices

In general, we found teachers’ use of specific instructional and behavior management practices was comparable across time for the majority of strategies measured by the CSAS. This is not surprising given that the observations in current study were non-evaluative and were not conducted as part of routine performance evaluations. Therefore, there was no expectation placed on teachers to change their classroom practices. Furthermore, it was not the direct intention of the current study to measure sensitivity to change following feedback, but to assess the utility and usability of the CSAS in the Greek context for informing potential changes to teachers’ classroom practices and professional development.

In light of this after three rounds of observations and feedback, we did find significant increases in the strategy of Academic Praise and significant improvement in using the strategies Promotes Students’ Thinking and Corrective Feedback Relatedly, although the majority of CSAS strategies did not change over time significantly, some immediate and positive changes in teachers’ instructional and behavioral management practices were observed.

The current study’s findings are in congruence with studies which also revealed positive results in teachers’ practices introducing low-intensity consultation procedures (O’Handley et al., 2018). For example, the increase in Academic Praise and Academic Response Opportunities found in our study was in agreement with Stitcher and colleagues’ study (2009), in which peer coaching increased the academic praise and opportunities to respond, although, opportunities to respond were increased to a lesser extent, maybe because they are more context dependent practices and require more than simply a prompt by a coach to improve, increase and maintain their use (Cavanaugh, 2013).

Our study shows contrasting results with studies using similar coaching procedures in teachers’ Behavior Praise. Although these studies revealed increase in teachers’ use of behavior praise (Cavanaugh, 2013; Briere et al., 2015; Simonsen et al., 2017), the current study did not reveal a significant increase in Behavior Praise usage. This may be due to this strategy’s low frequency of use. In our study, it is unclear why Behavior Praise rates declined, yet being lower from the initial observation. This latter finding though balances with the decrease found in the frequency of Corrective Feedback, that is teachers may have not improved their use of Behavior Praise, but they significantly improved the use of Corrective Feedback. In contrast, similar studies revealed no change in teachers’ use of reprimands (Corrective Feedback in CSAS; O’Handley et al., 2018).

There were specific practices, which although their frequency rates (i.e., Concept Summaries, Academic Response Feedback) or the quality rates (i.e., Adaptive Instruction, Student-Directed Instruction) in the third observation remained higher than the rates in the first observation, these rates actually decreased following the performance feedback of the second observation. These patterns of implementation with high, stable levels of intervention fidelity initially and decreased to lower levels after two observations are consistent with previous research (Sterling-Turner et al., 2002), indicating that without comprehensive training, teachers may require implementation support to increase levels of intervention fidelity within the first 2 weeks of intervention delivery (Collier-Meek et al., 2017). Mashburn et al. (2014) also indicated variation in teacher practices across a school day, due to either the attributions of the observational method, or the contextual variables within teachers.

There are several explanations for the lack of or the maintenance of change during the observations: First-based on some teachers’ comments-students in the classroom observed engaged in high levels of on-task and low-levels of disruptive behavior. Therefore, students’ behavior may not have been in need of implementing specific practices. Second, the emphasis given to certain skills during the brief performance feedback meetings may not have promoted the adequate implementation of all classroom management skills. The low dosage of performance feedback may also be a reason for our contrasting findings. Maybe teachers did not maintain the improved skill when they shifted their focus to a different skill. Teachers may have focused on implementing one skill rather than the full array of management skills.

Lastly, the school administrators’ scores on the CSAS, suggest classroom behavior management as an area that Greek teachers may struggle with. Specifically, our study found low usage of Behavioral Praise, which is a research supported strategy for proactively promoting positive behaviors in the classroom (Nagro et al., 2019). In comparison, there was a much higher usage of Behavioral Corrective Feedback, which is a reactive consequence strategy. In fact, there is almost a 1 to 7 ratio on average between Behavioral Praise and Behavioral Corrective Feedback. This contrasts with the long-standing recommendation from classroom management literatures supporting a positive 3 to 1 and 5 to 1 ratios in general education and special education contexts, respectively (Dudek et al., 2019). Related to the idea that teachers struggle with classroom behavior management, the observed teachers interviewed reported that they mainly received feedback from their observers regarding the use of Behavior Praise, and they admitted that they need additional training on classroom management practices. Perhaps, these practices may progress more slowly in coaching, or may be more difficult to be implemented in practice since they are independent or universal to all lessons, whereas instructional practices are more contextually dependent on the subject been taught (Reddy et al., 2017). In contrast, instructional practices required less implementation change than the behavior management practices. And this finding was also in congruence with the interview data, where teachers reported that CSAS feedback was particularly helpful with the use of instructional practices. Perhaps, instructional practices included practices which teachers were already using or were aware of, and they needed to be refined with the specific context.

### Limitations

The present study includes several limitations. First, findings from this investigation included a small sample of volunteer teachers, predominantly Greek females. Second, it is possible that school administrators’ classroom observations may have influenced teachers’ or students’ behavior which warrants investigation. Research on performance feedback has revealed negative results, for teachers with low self-efficacy (Bandura and Cervone, 1983), or when teachers or school administrators are over-or under-emotionally aroused based on the feedback (Kluger and DeNisi, 1996). Third, the present study did not include a control group. Fourth, we did not examine the long-term impact of feedback on teacher practices. Thus, future research should include assessment of maintenance of instructional and behavior management practices over a period of time, if we want to evaluate the maintenance of feedback gains. Fifth, teachers in the current study were not selected having challenges or deficits in instructional and/or behavior management practices. Finally, this study did not investigate teachers’ change in classroom practices and student outcomes. Future research would also employ student-focused observation measures to capture changes for both teachers and student outcomes.

### Implications

This study examined teachers’ instructional and behavior management implementation practices following a brief performance feedback. Results are promising albeit preliminary. These results could inform school administrators or consultants in designing their coaching activities, and in collaboration with teachers to identify an approach to support teachers with instructional and behavior management practices. The current study adds to the literature by suggesting instructional coaching as an effective form of PD that can support teachers’ use of evidence-based classroom practices within their classroom context. Instructional coaching PD programs embedded with performance feedback provide a promising method for supporting teachers’ implementation of classroom practices and bridging the implementation gap (Borko et al., 2011; Reinke et al., 2015; Dudek et al., 2019).

Studies point to the continued and persistent need for classroom management training (Stough et al., 2015). Darling-Hammond et al. (2017) described the importance of “sustained duration” PD. Our findings support and extend existing research on the need of classroom management training for teachers, and how the timely provision and specific performance feedback (Milanowski and Heneman, 2001; Eisenhart and Towne, 2003) can facilitate these changes. At this point, an evaluation of the dosage and performance delivery need to be examined to determine the most efficient dosage for teachers. It is argued that more comprehensive support brings effective teacher change (Fallon et al., 2019). However, low-intensity feedback report employed in our study revealed positive trends in teachers’ use of effective classroom management practices as well.

It is important to acknowledge the complexity of teaching and the increasing demands teachers face. It is not enough to tell teachers to be proactive or use praise to promote students’ positive behavior. Teachers need to have opportunities to engage with research in meaningful ways and experience the implementation of effective research-based practices (Hepburn and Beamish, 2019). It is important to evaluate teachers’ preferences or beliefs to determine if certain strategies are valued (Fullan, 2001; Fallon et al., 2019). These preferences might in turn provide better adherence to the intervention and feasibility of the intervention approach.

Finally, variability in teachers’ responses is anticipated (O’Handley et al., 2018), as individual teachers may respond differently to different coaches or different forms of feedback (Sprick et al., 2010), or duration of the PD (Desimone and Stuckey, 2014). Additional research is needed to better understand teachers’ experiences with instructional coaching and therefore increase the feasibility of the coaching. Besides the knowledge and skills instructional coaches need to have, according to Greek teachers, coaches need to be trusted (Kluger and DeNisi, 1996) and be “one of them” (Gravani and John, 2005).

## Conclusion

This study is the first step in examining teachers and school administrators’ participation in a small-scale PD schema, as measured by the CSAS. Findings were promising for the implementation of teachers’ instructional and behavior management practices following a brief performance feedback. The approach in this study was formative and collaborative suggesting that brief PD coaching models may support teachers’ implementation of evidence-based instructional and behavior management practices. Research is warranted that examines how PD efforts may support teachers’ implementation of universal evidence-based instructional and behavior classroom management strategies, under what conditions PD may support teachers taken into consideration teachers’ needs, and with which formats of PD the effective classroom management practices gained could maintain and generalized to other skills and students.

## Data availability statement

The raw data supporting the conclusions of this article will be made available by the authors, without undue reservation.

## Author contributions

MP conduct the research and writing. Linda Reddy had the research design, editing and CD did the statistical analysis and proof reading. All authors listed have made a substantial, direct, and intellectual contribution to the work and approved it for publication.

## Funding

This work was supported by the Society for the Study of School Psychology and International School Psychology Association.

## Conflict of interest

The authors declare that the research was conducted in the absence of any commercial or financial relationships that could be construed as a potential conflict of interest.

## Publisher’s note

All claims expressed in this article are solely those of the authors and do not necessarily represent those of their affiliated organizations, or those of the publisher, the editors and the reviewers. Any product that may be evaluated in this article, or claim that may be made by its manufacturer, is not guaranteed or endorsed by the publisher.
